# Environmental modulation of exopolysaccharide production in the cyanobacterium *Synechocystis* 6803

**DOI:** 10.1007/s00253-023-12697-9

**Published:** 2023-08-08

**Authors:** Mary Ann Madsen, Stefan Semerdzhiev, Jordan D Twigg, Claire Moss, Charles D Bavington, Anna Amtmann

**Affiliations:** 1 School of Molecular Biosciences, College of Medical, Veterinary, and Life Sciences, University of Glasgow, Glasgow G12 8QQ, Scotland, UK; 2 GlycoMar Ltd, Malin House, European Marine Science Park, Oban, Scotland, PA37 1SZ UK

**Keywords:** Cyanobacteria, Extracellular polymeric substances, Exopolysaccharides, Released polysaccharides, Nutrient limitation, Transcriptomic analysis

## Abstract

**Abstract:**

Microorganisms produce extracellular polymeric substances (EPS, also known as exopolysaccharides) of diverse composition and structure. The biochemical and biophysical properties of these biopolymers enable a wide range of industrial applications. EPS from cyanobacteria are particularly versatile as they incorporate a larger number and variety of building blocks and adopt more complex structures than EPS from other organisms. However, the genetic makeup and regulation of EPS biosynthetic pathways in cyanobacteria are poorly understood. Here, we measured the effect of changing culture media on titre and composition of EPS released by *Synechocystis* sp. PCC 6803, and we integrated this information with transcriptomic data. Across all conditions, daily EPS productivity of individual cells was highest in the early growth phase, but the total amount of EPS obtained from the cultures was highest in the later growth phases due to accumulation. Lowering the magnesium concentration in the media enhanced per-cell productivity but the produced EPS had a lower total sugar content. Levels of individual monosaccharides correlated with specific culture media components, e.g. xylose with sulfur, glucose and N-acetyl-galactosamine with NaCl. Comparison with RNA sequencing data suggests a Wzy-dependent biosynthetic pathway and a protective role for xylose-rich EPS. This multi-level analysis offers a handle to link individual genes to the dynamic modulation of a complex biopolymer.

**Key points:**

• *Synechocystis exopolysaccharide amount and composition depends on culture condition*

• *Production rate and sugar content can be modulated by Mg and S respectively*

• *Wzy-dependent biosynthetic pathway and protective role proposed for xylose-rich EPS*

**Graphical Abstract:**

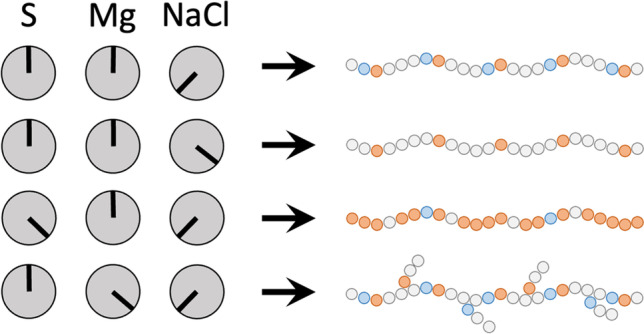

**Supplementary Information:**

The online version contains supplementary material available at 10.1007/s00253-023-12697-9.

## Introduction

Extracellular polymeric substances (EPS, also referred to as exopolysaccharides) are complex glycans secreted by various microorganisms including bacteria, microalgae and fungi (Freitas et al. 2017). These structurally diverse, high molecular weight polymers serve many functions in nature including protection against environmental stresses (starvation, desiccation, radiation, predation), interactions (surface adhesion, colony/biofilm formation, symbiosis), motility and infectivity (Costa et al. 2018; Kehr and Dittmann 2015; Rossi and De Philippis 2015).

Cyanobacterial EPS are more complex than those produced by other organisms. Depending on species and condition, they contain up to 13 different sugars as well as different modifications (anionic, hydrophobic and peptidic moieties), branching and linkage types (De Philippis et al. 2001; Panoff et al. 1988; Pereira et al. 2009). This diversity in EPS building blocks translates into tremendous structural complexity and functional variation which has enabled cyanobacteria to colonise some of the most extreme habitats on the planet (Bhatnagar and Bhatnagar 2019; Rossi and De Philippis 2015). The anionic, sulphated, or amphipathic characteristics of cyanobacterial EPS also result in chelating, emulsifying, gelling and immuno-modulating properties thus enabling a wide range of applications in food and healthcare (pharmaceutical, nutraceutical, cosmetics) or in wastewater and construction industries (De Philippis and Vincenzini 1998; Pereira et al. 2019b).

Not only are cyanobacterial EPS promising products, but the photosynthetic cyanobacteria themselves are also promising production platforms as light-powered cell factories (Camsund and Lindblad 2014; Cassier-Chauvat et al. 2021; Santos-Merino et al. 2019). In terms of manufacturing EPS, the most cost effective and energy efficient EPS product to harvest are the released exopolymeric substances (RPS) secreted into the surrounding environment, which maintain no or only very loose linkage to the cell surface. RPS can either be readily harvested in bulk from batch cultures at the end of the growth period or regularly ‘milked’ from the culture media of continuous cultivation systems (Cruz et al. 2020; Zydney 2016). It is therefore convenient that the model cyanobacterium *Synechocystis* sp. PCC 6803, in which the greatest engineering efforts have been made so far, maintains high EPS production rates in the late growth phase and into stationary phase (Panoff et al. 1988). Furthermore, EPS influence cell flocculation properties and can be used to enhance cell/media separation with no effect on cell viability thus enabling retention of the total biomass for long-term EPS production (Allen et al. 2019; Sun et al. 2020).

Three main pathways for EPS assembly and export have been found in bacteria and seem to be well conserved: Wzy-, ATP-binding cassette (ABC) transporter-, and synthase-dependent pathways (Pereira et al. 2009; Schmid 2018). EPS production starts with synthesis and activation of the sugar residues in the cytoplasm followed by assembly and polymerisation of sugar repeat units at the plasma membrane and final export across the outer membrane. Genes involved in EPS production can be categorised into three groups: 1) pathway genes for general sugar metabolism and not specific to EPS biosynthesis, 2) transferase genes for linkage of specific sugar residues, and 3) saccharide-processing genes for assembly, polymerisation, and export of EPS (Reeves et al. 1996). The modular nature of EPS biosynthesis is well suited to synthetic biology approaches: components of the pathways could be assembled and re-shuffled to adjust the types, order and frequency of sugars incorporated, as well as modifications, linkages, and branching, thus generating bespoke polymers with desirable properties (Pereira et al. 2019b). To develop this approach, it is paramount to understand the precise link between individual genes, enzymatic functions and secreted products.

Cyanobacterial genomes contain genes for all core functions of the EPS production machinery but, in contrast to the well-organised operons of other bacteria, EPS-related genes in cyanobacteria occur as multiple copies scattered throughout the genomes (Kehr and Dittmann 2015; Pereira et al. 2009; Pereira et al. 2015). Assignment of genes and assembly of complete pathways have therefore remained challenging. Initial investigations in the model cyanobacterium *Synechocystis* sp. PCC 6803 have confirmed the involvement of Wzy and ABC transporter pathways in EPS production (Fisher et al. 2013; Jittawuttipoka et al. 2013) and a biosynthetic pathway has been identified for the sulphated EPS synechan (Maeda et al. 2021). There is also good evidence that EPS production is regulated at the transcriptional level through the action of tyrosine and histidine kinase phosphorylation systems and alternative sigma factors such as SigF (Flores et al. 2019; Maeda et al. 2021; Pereira et al. 2019a).

EPS production often increases under stress. It is likely that, under conditions that limit biomass production, EPS synthesis provides a sink for excess electrons and photosynthetically assimilated carbon. For example, EPS production in several cyanobacterial strains was reported to increase under nutrient limitation (N, P, S, Mg, Ca) (De Philippis et al. 1991; Kharwar et al. 2021; Myklestad 1995) and oxidative stress (Hu et al. 2022), and in response to various abiotic factors including light quality and intensity, temperature, pH, salinity, and aeration (Bhatnagar and Bhatnagar 2019; Delattre et al. 2016). However, little is known whether environmental factors also alter the final composition of EPS. Monitoring the dynamic characteristics of the released polymer and integrating this information with changes in gene expression could potentially help to unravel the biosynthetic pathways. In this study, we therefore investigate how EPS change in response to different environmental conditions in the model cyanobacterium *Synechocystis* sp. PCC 6803. In the first part of the study, we evaluated factors that are of practical importance; we measured RPS production (titre and composition) in different growth phases of the cultures and we confirmed that they were not toxic for mammalian cells. In the second part of the study, we compared RPS production of cultures grown in different media (control, low S, low Mg, and 300 mM NaCl) and we integrated the data with previously obtained transcriptomics data for the same conditions. This multi-level analysis revealed novel correlations between environment, EPS sugar usage and genes, thus offering a basis for manipulating EPS composition in a model cyanobacterium.

## Materials and methods

### Culture conditions

*Synechocystis* sp. PCC 6803 was used in all experiments apart from the glucose experiments, which were carried out with the glucose-tolerant strain *Synechocystis* sp. PCC 6803-GT. Cultures were grown at 30°C with photoperiod 18-h/6-h light/dark, light intensity 120 ± 15 μmol photons/m^2^/s and sparged with humidified ambient air. 20 ml cultures were set up from glycerol stocks maintained at -80°C and gradually scaled up to 1.5 L cultures in Bijou bottles with a working volume of 60–75% of the bottle capacity. Cultures were grown in full BG11 medium (Stanier et al. 1971) for control conditions, in BG11 with 12.5% of the specified nutrients for low nutrient conditions (Madsen et al. 2021) or in BG11 with 300 mM NaCl or glucose added. For low light conditions, light intensity was reduced to 35 or 80 μmol photons/m^2^/s. Growth was monitored by measuring optical density at 730 nm (OD_730_) in a Lambda 45 UV/VIS Spectrophotometer (PerkinElmer, Waltham, MA, USA). To ensure measurements were performed in the linear range of the spectrophotometer, cultures were diluted in fresh media to OD <1, the sample OD was measured, and the culture OD was calculated by multiplying the sample OD with the respective dilution factor.

### RPS harvest

EPS harvest was performed at various times during culture growth to reflect different growth phases: 1) “early” at the beginning of rapid growth phase after initial lag phase, 2) “late” at the end of the rapid growth phase when culture growth slows down due to emerging limitations, and 3) “stationary” when culture density no longer increased, or decreased. The harvest days to reflect these phases were decided for each culture based on preliminary growth curves and regular OD measurements during growth. ODs at harvest varied because growth differed between cultures and conditions. At the end of the experiment only those samples that could be assigned to one of the three growth phases were included in the analysis. Details on harvest days and ODs are provided in supplemental Table S1 and supplemental Figure S1.

For RPS harvest, supernatant of cultures was collected by centrifugation at 4000 *g* for 10 min at 4 °C. The supernatant was vacuum filtered through a 1.2 μm cellulose ester filter (Sigma-Aldrich, St. Louis, MO, USA) and subsequently dialysed in 8 kDa cellulose dialysis tubing (Thermo Fisher Scientific, Waltham, MA, USA) against ELGA water (1:10) for 48 h with five water changes and constant slow stirring. The dialysed product was lyophilised at -60 °C and below 20 mTor pressure using a VirTis sentry 2.0 freeze dryer (SP Industries, Warminster, PA, USA). Dry RPS samples were weighed, and this value was used to determine RPS production rates. Water-soluble RPS stocks were suspended at 10 mg/ml in ELGA water and insoluble precipitates were removed by centrifugation at 8000 *g* for 2 min at 21 °C.

### RPS production analysis

The area under the growth curve (AUGC) was used to normalise RPS production titres to the total number of cells that were available for production up to the time point of harvest. It was calculated using Eq. 1:

$$\textrm{AUGC}=\sum \frac{\left({OD}_n+{OD}_{n-1}\right)}{2}\ast \left({D}_n-{D}_{n-1}\right)$$

where *OD* is the culture density as optical density at 730 nm, *D* is the culture age in days, and *n* represents day of OD measurement. This equation approximates the area under the growth curve with the sum of areas of rectangles where one side is time between OD measurements and the other side is the linear average of two consecutively measured ODs. In an alternative approach we tried to fit established growth models (e.g. Gompertz) to the measured ODs to calculate integrals. However, goodness of fits varied between cultures, and we therefore decided to use Equation 1 as the simplest approximation for all cultures based on the measured OD values.

### RPS compositional analysis

Protein concentration was determined using a bicinchoninic acid (BCA) assay with bovine serum albumin (BSA) as reference (Thermo Fisher Scientific). Carbohydrate concentration was determined using the phenol-sulfuric acid method with glucose as reference (Dubois et al. 1956). Sulphate concentration was determined using the sodium rhodizonate assay with H_2_SO_4_ as reference (Terho and Hartiala 1971).

Monosaccharide composition was determined by methanolysis/tri-methylsilane derivatisation followed by quantitative analysis using a Shimadzu GC-2014 Gas Chromatograph equipped with a Flame-Ionisation Detector, Zebron ZB-5 MS column, Shimadzu FocusLiner (Shimadzu, Kyoto, Japan) and 300 °C splitless injection of 1 μl samples. References were included for each of the reported monosaccharides (Sigma-Aldrich).

High-performance liquid chromatography (HPLC) - size exclusion chromatography (SEC) was performed using an Alliance 2695 HPLC system equipped with a Shodex SB806M aqueous GFC column (Shodex) and refractive index detector (Waters, Milford, MA, USA) with dextran and heparin as references (Sigma-Aldrich). The mobile phase was 0.1 mM EDTA, 5 mM Tris pH 7, 0.09% NaCl at a flow rate of 0.5 ml/min.

### RNA-sequencing

The transcriptomics experiment was previously published by our group (Madsen et al. 2021) and the sequencing dataset is available from the European Nucleotide Archive (PRJEB40560). In brief, mRNA was harvested from two growth phases (early and late) of *Synechocystis* spp. PCC 6803 cultures grown in control (BG11) and five nutrient limited conditions (12.5% N, P, K, Mg or S in BG11 background) in three biological replicates (independently grown cultures). Data are presented as fragments per kilobase of gene per million reads mapped (FPKM). Significant differences between conditions and time points were determined using Cuffdiff (Trapnell et al. 2012).

### Statistical analyses

Statistical analysis of multiple comparisons was carried out in SigmaPlot software using one-way ANOVA with Tukey post hoc analysis, or Kruskal-Wallis One Way Analysis of Variance on Ranks with Dunn’s post-hoc analysis, a non-parametric test that does not require normal distribution. Pearson correlation analysis for multicomponent comparisons was performed in R software, using the cor.test function.

## Results

### Released extracellular polymeric substances (RPS) in different growth phases

#### RPS production rates in different growth phases

**Fig. 1:**
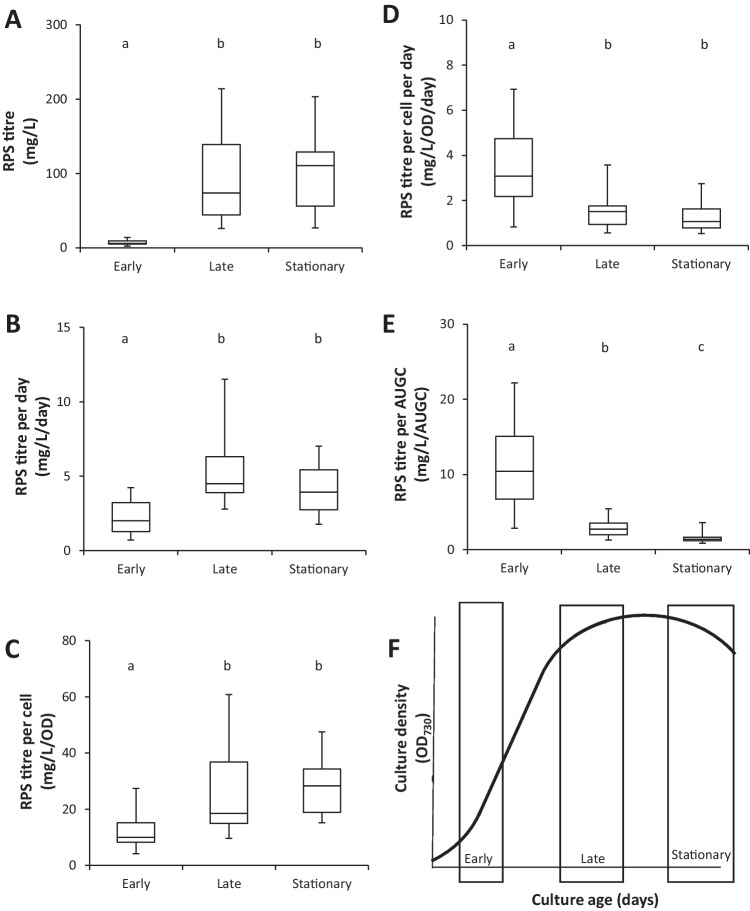
RPS production in different growth phases. RPS production is expressed as (**A**) dry weight per culture volume (titre), (**B**) titre per day, (**C**) titre per cell (using OD_730_ as a proxy for cell density), (**D**) titre per cell per day, and (**E**) titre per area under the growth curve (AUGC, see Eq. 1). RPS was harvested by dialysing and lyophilising the supernatant of *Synechocystis* sp. PCC 6803 cultures in three different growth phases: early growth (*n*=17), late growth (*n*=23) and stationary phase (*n*=14). Different letters indicate significant difference at adjusted *p*-values of *p*<0.005 (a,b) and *p*<0.05 (c) as determined by Kruskal-Wallis ANOVA with Dunn’s post hoc multiple comparison analysis. Harvest periods in a schematic growth curve are shown in (**F**). Cultures were grown in a range of conditions. Information on conditions, growth curves and harvest days for each culture are provided in Table S1

To compare RPS production at different stages of culture growth, RPS samples were harvested by dialysing and lyophilising the supernatant of batch cultures. A total of 54 samples were obtained during the “early” exponential, “late” transition to stationary, and “stationary” growth phases from cultures grown in a range of different media including BG11 (control), BG11 with low (12.5%) concentrations of N, K, P, Mg or S (see Madsen et al. 2021), and BG11 with NaCl or glucose added, as well as low light conditions. Information on all samples, ODs and harvest days is provided in Table S1. The variety of cultures used for this analysis was intentionally broad to extract consistent differences between growth phases. Figure 1 shows RPS production in the different growth phases. RPS titre (dry weight per culture volume; mg/L, Fig. 1a), daily productivity (titre normalised to culture age in days; mg/L/day, Fig. 1b) and RPS titre per cell (titre normalised to OD, mg/L/OD, Fig. 1c) were low in early growth, increased in late growth and remained high in the stationary phase. These measures indicate that synthesis of RPS is faster than degradation and therefore RPS accumulate in the culture media of batch cultures. However, the values obtained for daily RPS titre per cell (mg/L/OD/day, Fig. 1d) and for titre over area under the growth curve (mg/L/AUGC, Fig. 1e) show that the net daily RPS production rates per cell are highest in the early growth phase followed by significant decreases in later growth phase. These results indicate that *Synechocystis* cells are more productive during early growth compared to the later growth phases (for assignment into growth phases see Fig. 1f and supplemental Table S1). In other words, if the initial production rates were maintained over the entire growth period the final titre of RPS would be expected to be higher than the measured value. This has implications for larger scale production where continuous milking of a continuous culture in early exponential phase could potentially achieve higher cumulative yields than harvest from batch culture in stationary phase.

#### RPS composition in different growth phases

**Fig. 2:**
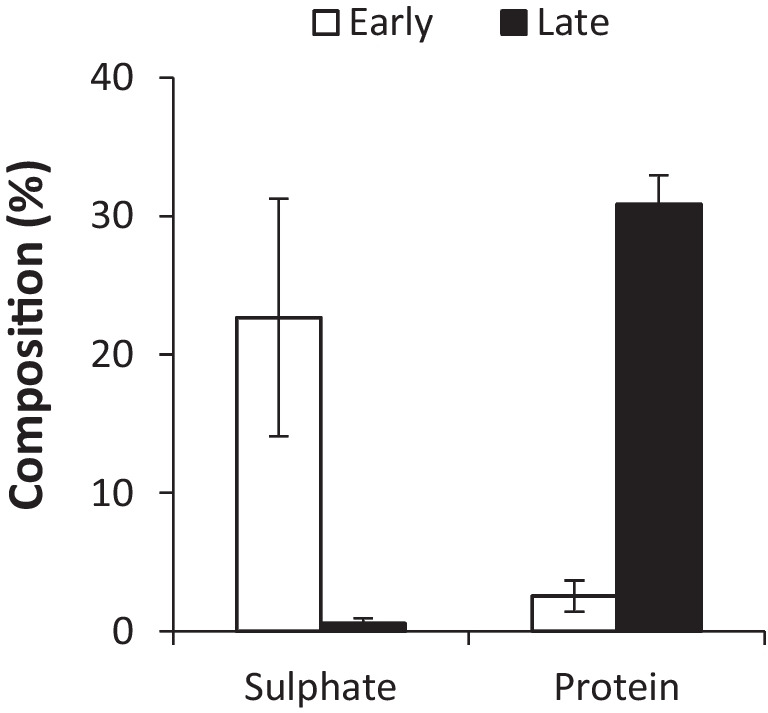
RPS composition in different growth phases. Sulphate and protein content of water-soluble RPS harvested during early and late growth of *Synechocystis* sp. PCC 6803 cultivated in control BG11 media. Sodium rhodizonate and BCA assays were used to determine sulphate and protein, respectively. Data are means ± S.E.M. of three independent cultures

We next set out to compare the composition of water-soluble RPS between early and late *Synechocystis* sp. PCC 6803 cultures grown in control BG11 media. Water-soluble RPS were isolated from dry total RPS samples by resuspending in water (10 mg/ml) and removing insoluble particulate matter. EPS are complex polymers comprised of a sugar backbone decorated with various non-sugar components such as acidic and anionic side groups, proteins and DNA (De Philippis and Vincenzini 1998; Pereira et al. 2009). Of particular interest are sulphated EPS as they are associated with various bioactive and immunomodulatory properties (Raposo et al. 2013). Figure 2 shows that sulphate content (detected with a sodium rhodizonate assay) was higher in the early growth phase than the late growth phase with the latter being below the detection limit (<3.8% by weight). By contrast, protein content was low in the early growth phase and high in the late growth phase. EPS from early samples could not be detected by HPLC-SEC and generated poor GC-FID signals. Late samples generated complex HPLC-SEC profiles with EPS of varying sizes and good GC-FID signals as described in detail below. Combined, the ability to identify EPS in late but not early samples, despite starting with the same amount of dry total RPS material, suggests differences in solubility and/or amenity to acid hydrolysis and therefore differences in the content and/or structure of RPS produced during different stages of growth.

#### Effect of cyanobacterial RPS on mammalian cells

**Fig. 3:**
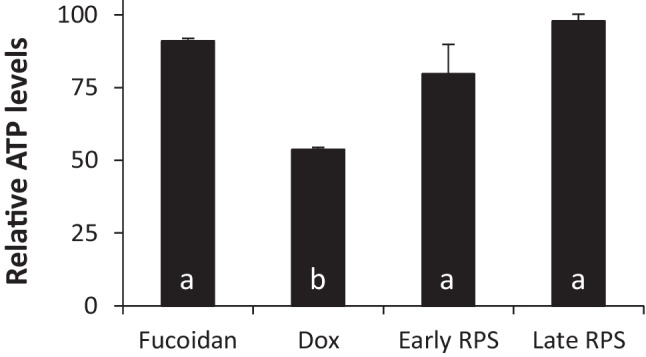
Metabolic activity of mammalian cells treated with RPS. ATP levels (relative to the HBSS vector control) after 24 h treatment of mammalian baby hamster kidney (BHK) cells with water-soluble RPS harvested during early and late growth of *Synechocystis* sp. PCC 6803 cultivated in control BG11 media. Fucoidan serves as negative control showing low cytotoxicity. Doxorubicin hydrochloride (Dox) serves as positive control showing high cytotoxicity. Data are means ± S.E.M. of three technical replicates from two independent cyanobacterial cultures. Different letters indicate significant difference (*p*<0.05; one-way ANOVA with Tukey post hoc analysis)

For potential applications in health-related industries, it is important to assess whether cyanobacterial EPS samples are safe for use in mammalian cells. Figure 3 shows the metabolic activity (as ATP levels) of baby hamster kidney (BHK) cells treated with water-soluble RPS samples isolated from *Synechocystis* cultures grown in control conditions. RPS samples harvested during early or late growth phases had little impact on the metabolic activity of BHK cells. While relative ATP levels were slightly lower in cells treated with early RPS samples than in cells treated with late RPS samples, they were similar to ATP levels in cells treated with fucoidan, a sulphated polysaccharide with low cytotoxicity (Nagaoka et al. 2000), and significantly higher than in cells treated with doxorubicin hydrochloride (Dox), an anthracycline antibiotic with high cytotoxicity (Thorn et al. 2011). BHK cells treated with late RPS samples showed a slightly higher ATP level than those treated with fucoidan. In summary, the water-soluble RPS of *Synechocystis* sp. PCC 6803 is not toxic to mammalian cells independent of whether they are isolated from early or late-stage cultures.

### RPS production in different environmental conditions

Cyanobacterial EPS production often increases under environmental stress (Delattre et al. 2016; Myklestad 1995). To identify nutritional conditions that may alter EPS production, we mined a previously obtained transcriptomics dataset of *Synechocystis* sp. PCC 6803 grown in different media (Madsen et al. 2021) for genes with published function in EPS biosynthesis. Three genes of a Wzy-dependent pathway (Jittawuttipoka et al. 2013) showed opposite responses during the transition to the late growth phase with up-regulation in low Mg (Fig. S1a) and down-regulation in low S (Fig. S1b). We therefore used cultures grown in BG11 with 12.5% of the original Mg or S concentration (low Mg, low S) for further EPS analyses. We also included samples grown with high salinity (BG11 with 300 mM NaCl added) as increased EPS production under salt stress and its requirement for salt tolerance had been reported by others (Ozturk and Aslim 2010) (Jittawuttipoka et al. 2013). Fig. S2 shows growth curves of the cultures and photographs of dry RPS samples from the different cultures. RPS samples were harvested in late exponential phase.

#### RPS production rates in different environmental conditions

**Fig. 4:**
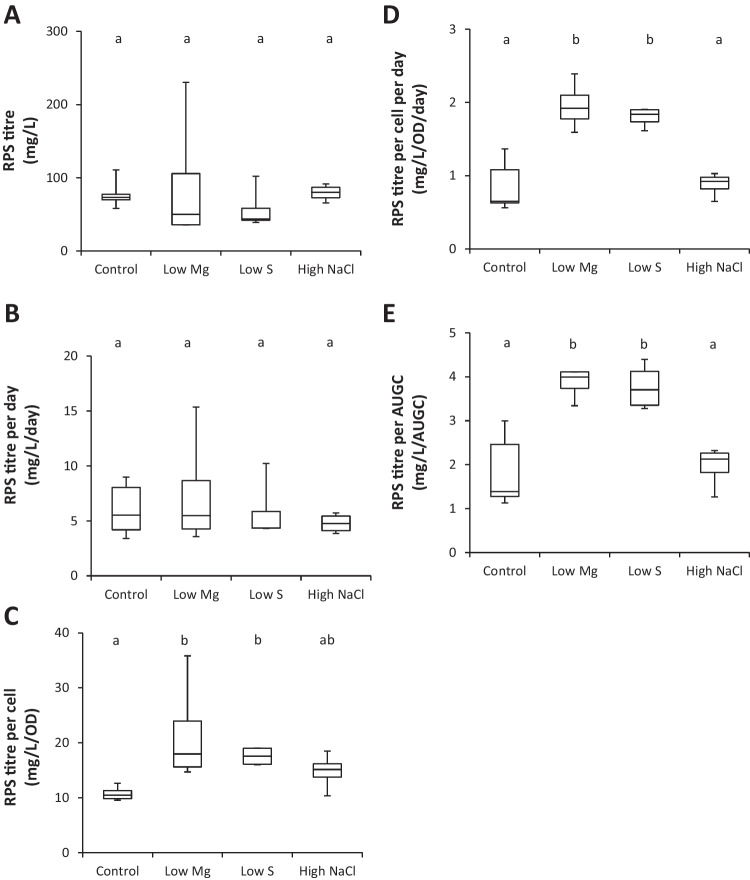
RPS production rates in different media. Total RPS of *Synechocystis* sp. PCC 6803 grown to the late growth phase in control (*n*=6), 12.5% Mg (*n*=4), 12.5% S (*n*=4) and 300 mM NaCl (*n*=4) in BG11 background. RPS production is expressed as (**A**) dry weight per culture volume (titre), (**B**) titre per day, (**C**) titre per cell (using OD_730_ as a proxy for cell density), (**D**) titre per cell per day, and (**E**) titre per area under the growth curve (AUGC, see Eq. 1). Different letters indicate significant difference (*p*<0.05; one-way ANOVA with Tukey post hoc analysis)

Figure 4 shows RPS production rates in the different culture media as titre normalised to time and/or culture density. Total culture productivity, measured as RPS titre (mg/L, Fig. 4a) and RPS titre normalised to culture age (mg/L/day, Fig. 4b), showed similar RPS production rates across the different conditions. “Per cell” productivity obtained from titres normalised to culture density either alone (mg/L/OD, Fig. 4c) or in combination with culture age (mg/L/OD/day, Fig. 4d) and from area under the growth curve (mg/L/AUGC, Fig. 4e), showed significant increases under nutrient limitation (low Mg and low S) compared to the control condition. Adding salt (300 mM NaCl) to the culture medium did not improve RPS production. In summary, nutrient limitation enhances the productivity of individual cells, but overall productivity of the culture remains unchanged due to lower culture density compared to control.

#### RPS composition in different environmental conditions

**Fig. 5:**
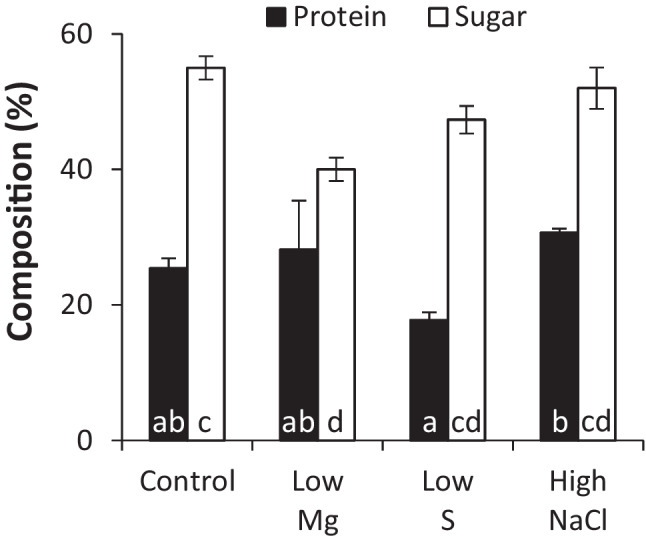
Protein and sugar content of RPS in different media. Protein (BCA assay, *n*=4) and sugar content (phenol-sulfuric assay, *n*=3) of water-soluble RPS from *Synechocystis* sp. PCC 6803 grown to the late growth phase in control, 12.5% Mg, 12.5% S and 300 mM NaCl in BG11 background. Data are means ± S.E.M. Different letters indicate significant difference (*p*<0.05; two-way ANOVA with Tukey post-hoc analysis)

**Fig. 6:**
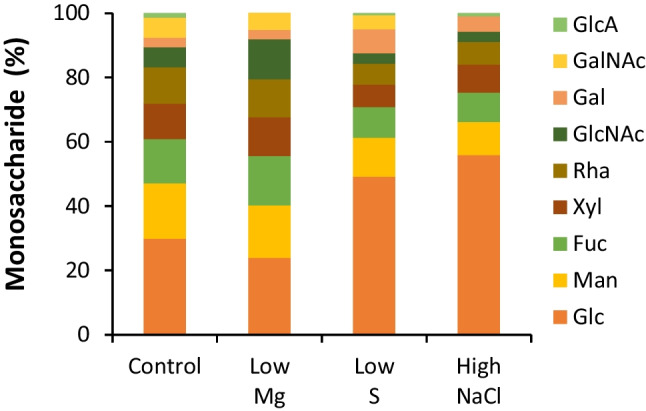
Relative monosaccharide content of RPS in different media. Average monosaccharide levels (relative to total recovery, GC-FID) of water-soluble RPS from *Synechocystis* sp. PCC 6803 grown to the late growth phase in control (*n*=5), 12.5% Mg (*n*=4), 12.5% S (*n*=3), and 300 mM NaCl (*n*=4) in BG11 background. Monosaccharides are Glc glucose, Man mannose, Fuc fucose, Xyl xylose, Rha rhamnose, GlcNAc N-acetyl-glucosamine, Gal galactose, GalNAc N-acetyl-galactosamine, GlcA glucuronic acid

**Fig. 7:**
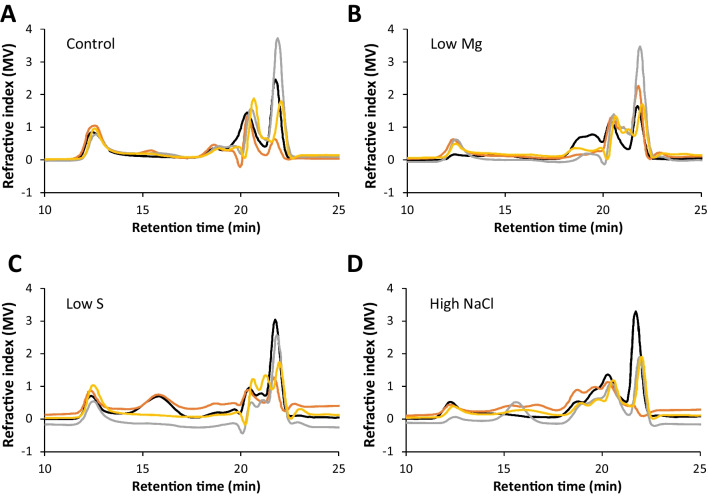
Molecular weight profiles of RPS in different media. HPLC-SEC refractive index chromatograms of water-soluble RPS from *Synechocystis* sp. PCC 6803 grown to the late growth phase in (**A**) control, (**B**) 12.5% Mg, (**C**) 12.5% S, and (**D**) 300 mM NaCl in BG11 background. Different colours represent independent cultures

We next characterised the composition of the water-soluble fraction of late RPS samples harvested from the different conditions. Figure 5 shows that the RPS produced in control media were mostly comprised of sugar (55%) and protein (25%). Sulphur content was below the detection limit of the sodium rhodizonate assay (<3.8%). Sugar content decreased significantly when Mg levels in the culture media were low. Figure 6 shows that, of the monosaccharides detected by GC-FID, there were six “core” monosaccharides present in all conditions: glucose was the most common followed by mannose, fucose, xylose, rhamnose and N-acetyl-glucosamine. Also common were N-acetyl-galactosamine and galactose, detected in 93.8 and 87.5% of samples respectively. Glucuronic acid was detected in 25% of samples. Arabinose, iduronic acid and galacturonic acid were not detected in any of the samples tested. Correlation analysis of 19 variables including culture media components, RPS composition, and RPS production rates shows a very strong correlation between four of the core monosaccharides (mannose, fucose, xylose and rhamnose) and N-acetyl-galactosamine, which were consistently present at high levels when glucose was low (Table 1). Decreasing Mg levels in the culture media strongly enhances the productivity of individual cells (mg/L/OD, mg/L/OD/day, mg/L/AUGC), but the RPS had a lower sugar content, suggesting a tradeoff between RPS production rate and total sugar content. Low S correlated with low protein and low xylose contents. High NaCl in the media resulted in higher levels of glucose and lower levels of N-acetyl-galactosamine. In fact, N-acetyl-galactosamine was detected in all conditions except high NaCl. The HPLC-SEC refractive index chromatograms in Fig. 7 show that all conditions produced very high molecular weight RPS, evident as a peak at the resolution limit of the column (≥5 MDa, ~12.4 min) with a broad shoulder representing a mixture of molecules outside of the resolution of the dextran standard (>1.4 MDa, <16 min). All conditions also produced a mixture of smaller RPS molecules (<36 kDa, >18.5 min) evident as multiple peaks prior to the buffer peak (~22 min). In some samples, a distinct, symmetrical peak occurred at 15.7 min, suggesting an increase in either a single molecule or a uniform mixture of molecules. The HPLC profiles showed some consistent differences, e.g. presence of peaks in the 20-22 min range of low S or high NaCl samples compared to control, but these fractions as well as the larger RPS would require additional separation for deconvolution. Further analytical techniques will be needed to link culture conditions with specific polymer structures.

**Table 1:**
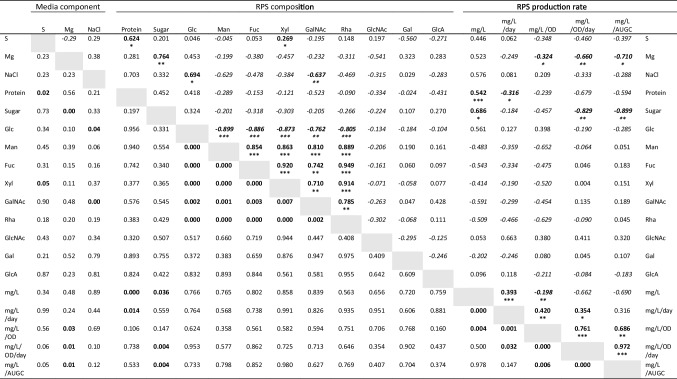
Pearson correlation analysis of RPS production under different environmental conditions

Each cell above/right of the grey boxes shows the correlation coefficient for positive or negative (italics) correlation. Statistically significant correlations are marked in bold and asterisks indicate *p*-values (*p<0.05, **p<0.01, ***p<0.001; calculated using a t-distribution with n-2 degrees of freedom). Exact *p*-values are given in the cells below/left of the grey boxes. Glc: glucose, Man: mannose, Fuc: fucose, Xyl: xylose, GalNAc: N-acetyl-galactosamine, Rha: rhamnose, GlcNAc: N-acetyl-glucosamine, Gal: galactose, GlcA: glucuronic acid. OD: optical density at 730 nm, AUGC: area under growth curve

#### Transcriptional analysis of RPS production

**Fig. 8:**
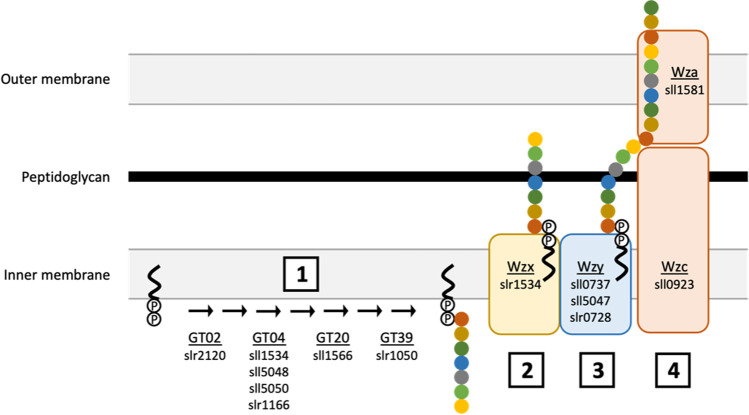
Proposed xylose rich RPS production pathway. Schematic diagram shows genes with strong correlation (*p*<0.05) to xylose content of water-soluble RPS from *Synechocystis* sp. PCC 6803. These genes encode EPS machinery for (1) assembly of repeat sugar units by glycosyltransferases, (2) transfer across the inner membrane by flippase Wzx, (3) polymerisation of repeat units by polymerase Wzy, and (4) transfer across the outer membrane by Wza/Wzc complex

The modularity of EPS biosynthetic pathways offers opportunities for bioengineering bespoke polymers (Pereira et al. 2019b), however this requires detailed knowledge of the EPS production machinery and the genes that encode them. In *Synechocystis* sp. PCC 6803, a few genes have been identified which underpin the production of specific EPS products e.g. synechan (Maeda et al. 2021) or the incorporation of specific EPS components e.g. fucose (Mohamed et al. 2005). In an attempt to assign functions to EPS-related genes, we performed Pearson correlation analysis comparing different parameters of RPS production (environmental conditions, production rates, composition) to RNAseq data for EPS-related genes (average gene expression level) during the late growth phase (Madsen et al. 2021). A list of 436 genes with annotated functions in EPS biosynthesis or carbohydrate metabolism was compiled from previous publications (Fisher et al. 2013; Flores et al. 2019; Maeda et al. 2021; Pereira et al. 2015) (Lombard et al. 2014). Table S2 lists the genes with normalised transcript levels and p-values of all pairwise comparisons (Madsen et al. 2021). The correlation analysis identified 260 and 110 genes with strong correlations (*p*<0.05) to total sugar and xylose content, respectively. The gene lists (with associated Pearson correlation and transcriptomic data) can be found in Table S3 for total sugar and Table S4 for xylose content. Table 2 shows the number of genes of a particular EPS-related functional annotation category in each gene list. Interestingly, Pfam domains associated with Wzy-dependent pathways are generally negatively correlated with total sugar content (Wzb, Wzc, Wzx, Wzy) and positively correlated with xylose content (Wza, Wzc, Wzx, Wzy). This suggests that Wzy-dependent pathways may be important for the integration of specific monosaccharides into EPS. Furthermore, this analysis points towards a Wzy-dependent biosynthetic pathway for xylose-rich RPS, summarised in Fig. 8. The proposed pathway is comprised of 7 glycosyltransferases (sll1534, sll1566, sll5048, sll5050, slr1050, slr1166 and slr2120), one Wzx flippase (slr1543), 3 Wzy polymerases (sll0737, sll5047, slr0728), and one outer membrane complex comprised of Wzc (sll0923) and Wza (sll1581). Some of the potentially regulatory genes that correlated with xylose content have previously been reported to be involved in stress responses and included 6 genes encoding histidine kinase sensor and/or response regulators responding to acid (slr1759, slr1909) (Chen et al. 2016; Michel et al. 2009; Nodop et al. 2006), heavy metal (sll0649) (Chen et al. 2014b), butanol (slr1037) (Chen et al. 2014a) and Ci stress (slr0312) (Wang et al. 2004), as well as hybrid sensor and regulator sll5060 of unknown function (Xu and Wang 2019). In summary, the correlation analysis suggests 1) a Wzy-dependent biosynthesis and export pathway and 2) a protective role for xylose-rich RPS.

**Table 2:** Numbers of genes with strong correlations to EPS features (*p*<0.05)

Abbreviation	EPS-related function	Total^a^	Total sugar content			Xylose		
			+^b^	-^c^	Total^d^	+^b^	-^c^	Total^d^
*Cell wall and cell surface macromolecular components*^1^								
App	Appendages	108	30	30	60	11	17	28
CW	Cell wall	75	19	27	46	9	11	20
LPSB	Lipopolysaccharide biosynthesis	40	18	17	35	3	2	5
PGS	Peptidoglycan synthesis	14	5	6	11	0	3	3
PSB	Polysaccharide biosynthesis	132	40	40	80	21	16	37
SMMS	Small molecular metabolite synthesis	51	19	10	29	4	10	14
*Carbohydrate active enzymes (CAZy)*^2^								
CBM	Carbohydrate-binding module	7	2	2	4	0	2	2
CE	Carbohydrate esterase	1	0	0	0	0	0	0
GH	Glycoside hydrolase	17	2	5	7	2	6	8
GT	Glycosyltransferase	72	19	29	48	7	8	15
*Pfam domains related to EPS production*^3^								
Alg44, BscA		2	1	0	1	0	0	0
Alg8, BscA		4	0	3	3	0	1	1
ExoD		1	0	1	1	0	0	0
KpsF	(Kdo linker synthesis)	9	3	5	8	1	0	1
KpsM (Wzm)	(ABC transporter)	5	3	1	4	1	0	1
KpsS, KpsC	(Kdo linker synthesis)	1	0	0	0	0	0	0
KpsT (Wzt)	(ABC transporter)	54	12	21	33	2	12	14
KpsU	(Kdo linker synthesis)	1	1	0	1	0	0	0
Wza, KpsD	Outer membrane polysaccharide export (OPX)	1	0	0	0	1	0	1
Wzb	Phosphatase, regulates Wzc	2	0	1	1	0	0	0
Wzc, KpsE (Wzz)	Polysaccharide copolymerase (PCP)	2	0	1	1	1	0	1
Wzx	Flippase	3	0	2	2	1	0	1
Wzy (WaaL)	Polymerase	5	1	1	2	3	0	4

^a^Total number of genes in the EPS-related functional annotation category

^b^Number of genes with strong positive correlation

^c^Number of genes with strong negative correlation

^d^Total number of genes with strong correlation

^1^Fisher et al. 2013, DOI:10.1371/journal.pone.0074514

^2^Lombard et al. 2014, DOI:10.1093/nar/gkt1178

^3^Pereira et al. 2015, DOI:10.1038/srep14835

## Discussion

Extracellular polymeric substances are versatile bio-based polymers that can offer sustainable alternatives for many industries. The modular nature of EPS biosynthesis has great potential for the rational design of biomaterials with new and bespoke properties (Pereira et al. 2019b). However, plug-and-play approaches require a comprehensive understanding of the biosynthetic genes, machinery and pathways. While genes involved in cyanobacterial EPS production have been identified, functional understanding of the encoded proteins and organisation into biosynthetic pathways is still very limited (Fisher et al. 2013; Maeda et al. 2021; Pereira et al. 2015). Given that cyanobacteria adjust EPS amount and composition to protect from changing conditions (De Philippis and Vincenzini 1998), we characterised RPS produced by a single cyanobacterium in different growth phases and culture media and compared with transcriptomic data to begin unravelling the genes underpinning EPS production and its regulation. We identified culture media components that modulate both the production rate and composition of RPS as well as a potential biosynthetic pathway for xylose-rich RPS in *Synechocystis* sp. PCC 6803.

### Responses to changes in growth phase

EPS production generally increases in the stationary phase (Delattre et al. 2016; Panoff et al. 1988). Indeed, we observed an increase in overall EPS production in the late growth and stationary phases of *Synechocystis* sp. PCC 6803 batch cultures (Fig. 1a). However, we found that the daily productivity of individual cells was in fact highest during the early growth phase (Fig. 1d, e). The decrease in cell productivity during the later growth phases was masked by the high cell density—a feature also observed in the efficient EPS producer *Cyanothece* sp. CCY 0110 where the total number of cells contributes more to overall productivity than the productivity of individual cells (Mota et al. 2013). Properties of RPS produced in the different growth phases also differed, evident in their amenability (or lack thereof) to different analytical techniques. Samples harvested during the early growth phase could not be detected by liquid or gas chromatography while late samples yielded strong signals, suggesting differences in RPS content, structure and/or physicochemical characteristics. Increasing culture volume, and therefore amount of dry RPS material harvested, could help to improve the resolution of early stage RPS products. While saccharide content could not be compared between growth phases in this study, analysis of sulphate content showed that sulphated RPS were only produced in the early growth phase (Fig. 2). Furthermore, we observed different levels of sulphation across one replicate of early RPS samples grown in different culture media (data not shown). In this study, however, we opted to focus on RPS harvested during the late growth phase given their amenability to more detailed compositional analysis and did not detect sulphate in any of the late samples examined. Sulphated RPS are associated with bioactive and immunomodulatory properties (Raposo et al. 2013), and we showed that the RPS of *Synechocystis* sp. PCC 6803 is not toxic to mammalian cells (Fig. 3). The RPS of *Synechocystis* sp. PCC 6803 is therefore a good candidate for the identification and production of new treatments for healthcare applications. A biosynthetic pathway for a sulphated RPS, synechan, was recently proposed for *Synechocystis* sp. PCC 6803 (Maeda et al. 2021). Comparison of sulphate content in early RPS samples with gene expression levels in different culture media would confirm the gene(s) underpinning sulphated RPS production and provide insights to the function and regulation of genes involved in the biosynthesis of synechan and other sulphated RPS. Protein content was found to be higher in the late growth phase than in the early growth phase (Fig. 2). This could be due to an increased N:C availability when N-supply in the media is still sufficient but C assimilation is limiting because of insufficient CO_2_ supply or shading (Kim et al. 2010).

### Responses to changes in the environment

Culture media offer a cheap and simple solution to control product synthesis in microbial factories. EPS are particularly amenable to this approach given their role in forming a dynamic shield whose amount and composition adjusts to suit changing conditions (De Philippis and Vincenzini 1998). In this study, we investigated just three media components (Mg, S and NaCl) and showed that all three components could be used to modulate RPS production in *Synechocystis* sp. PCC 6803. Firstly, productivity of individual cells was significantly improved under nutrient limitation (Fig. 4cc, d, e), particularly low Mg, and is consistent with the general role of the anionic EPS to capture and store essential nutrients in nature (De Philippis et al. 1991; Delattre et al. 2016). While decreasing levels of individual nutrients boosted “per cell” productivity, the concomitant decrease in culture density negated any improvements in overall productivity, i.e. productivity of the total biomass (Fig. 4a, b). The limited nutrients were supplied at a very low level in this study (12.5% relative to the control medium) leading to a large decrease in culture density (e.g. 50% in low Mg; Fig. S2). Fine tuning nutrient levels may achieve high cell productivity at a relatively high culture density to improve overall productivity for industrial manufacturing processes. Several other nutrients are also reported to affect EPS production in cyanobacteria including P, Ca, and N and could be optimised for RPS production (Qian et al. 2022; Singh et al. 2016; Zevin et al. 2015). It is important to note, however, that altering nutrient levels can lead to significant changes in saccharidic composition, both in terms of total sugar content (e.g. by Mg; Fig. 5) and the amount of individual monosaccharides (e.g. by S and NaCl; Fig. 6) present in the EPS (Table 1). The next step will be to identify which polymers are the main contributors to the observed changes. Note that there is no evidence that *Synechocystis* produces cellulose (Nobles et al., 2001) and unlike some other cyanobacteria (Zhao et al. 2015) its genome does not contain the CesA gene for cellulose synthase. In addition to media composition, other conditions known to affect EPS production in cyanobacteria could offer additional options to increase production, including light (intensity, duration, wavelengths), temperature, aeration and/or agitation (Delattre et al. 2016; Mota et al. 2013; Soule et al. 2016). EPS composition is also influenced by abiotic factors, and it is therefore essential to ensure that the optimised cultivation conditions do not compromise product integrity for industrial manufacturing.

### EPS production pathways

Biosynthetic pathways can be engineered to decouple product synthesis from other cellular processes, e.g. growth or stress response (Burg et al. 2016). Product integrity can thus be maintained irrespective of, e.g. culture media composition, by placing genes encoding EPS production machinery under the control of regulatory elements, i.e. promoters, that do not respond to changes in nutrient supply. Subsequent tweaking of expression levels of individual components can balance the flux within the EPS biosynthesis pathway, and wider metabolic engineering can channel resources/alleviate bottlenecks into this pathway to further improve productivity (Angermayr et al. 2015; Vijayakumar et al. 2020). Entirely new EPS biosynthetic pathways can be rationally designed by combining specific genes to determine EPS composition and structure thus creating bespoke polymers with selected properties (Pereira et al. 2019b). Alternatively, random combinations of sugars, modifications and linkages can be generated using gene shuffling approaches to generate new polymers and may in fact yield more genetically stable expression constructs for cyanobacteria (Taylor et al. 2021). However, pathway engineering requires detailed knowledge of EPS production machinery and its regulation. Cyanobacterial genes have been identified but functional annotation, biochemical characterisation, and assignment to biosynthetic pathways is limited (Fisher et al. 2013; Maeda et al. 2021; Pereira et al. 2015). In this study, we attempted to begin assigning specific functions to EPS-related genes by comparing RPS composition with transcriptomic data. While we did not identify genes underpinning specific EPS features, we did identify a candidate pathway for the production of RPS with high xylose content, summarised in Fig. 8. This may generate a product with repeating units of 7 monosaccharides assembled by 7 glycosyltransferases, one or more of which will presumably catalyse the transfer of “activated” xylose nucleotide monomers to the growing polysaccharide chain. This is similar to the 8 monosaccharide repeats assembled by 8 glycosyltransferases in synechan biosynthesis (Maeda et al. 2021). Repeat sugar units are then transferred across the inner membrane by a single flippase (Wzx), assembled into larger chains by up to three polymerases (Wzy) and exported by an outer membrane transport complex (Wza/Wzc). This pathway was originally characterized in *E. coli* (Whitfield and Paiment 2003) but has yet to be linked to specific polymeric products within cyanobacterial RPS. In *Synechocystis* 6803, this pathway includes 5 genes of unknown function (sll1534, slr0728, slr1050, slr1543, slr2120), including the Wzx flippase, which provide a good starting point for targeted knockout analyses.

To conclude; this study provides insights into how environmental conditions, notably culture media, can be used to control EPS production and composition in a cyanobacterium and further proposes a new EPS biosynthesis pathway. Characterisation of more EPS features in more environmental conditions, paired with “omics and knockout analyses,” will generate a more detailed picture of EPS biosynthesis, its regulation and the machinery involved. This knowledge base will improve industrial production and enable approaches using both random and carefully selected combinations of EPS production machinery to generate polymers with new and exciting properties for industry.

## Data Availability

All data generated or analyzed during this study are included in this published article and its supplementary information files. The RNAseq data is deposited at ENA, https://www.ebi.ac.uk/ena under project accession number PRJEB40560.

## Supplemental information

ESM 1 Fig. S1 RNA levels of selected genes in cultures grown in different growth media (PPTX 47 kb) ESM 2 Fig. S2 Growth and RPS extracts of *Synechocystis* sp. PCC 6803 in different media (PPTX 212 kb) ESM 3 Table S1 Growth curves and RPS harvest days of *Synechocystis* cultures included in Figure 1. (XLSX 171 kb) ESM 4 Table S2 EPS-related genes of *Synechocystis* sp. PCC 6803 (XLSX 420 kb) ESM 5 Table S3 Genes with strong correlation to total sugar content of water-soluble RPS produced during the late growth phase by *Synechocystis* sp. PCC 6803 (XLSX 279 kb) ESM 6 Table S4 Genes with strong correlation to xylose content of water-soluble RPS produced during the late growth phase by *Synechocystis* sp. PCC 6803 (XLSX 132 kb)
