# Supplementary material for: Environmental modulation of exopolysaccharide production in the cyanobacterium Synechocystis 6803
Source: Appl Microbiol Biotechnol. 2023 Aug 8;107(19):6121–34. doi: 10.1007/s00253-023-12697-9 (PMC10485101; doi:10.1007/s00253-023-12697-9)
Supplement: ESM 1 — Fig. S1 RNA levels of selected genes in cultures grown in different growth media (PPTX 47 kb) [file 253_2023_12697_MOESM1_ESM.pptx]

## Slide 1
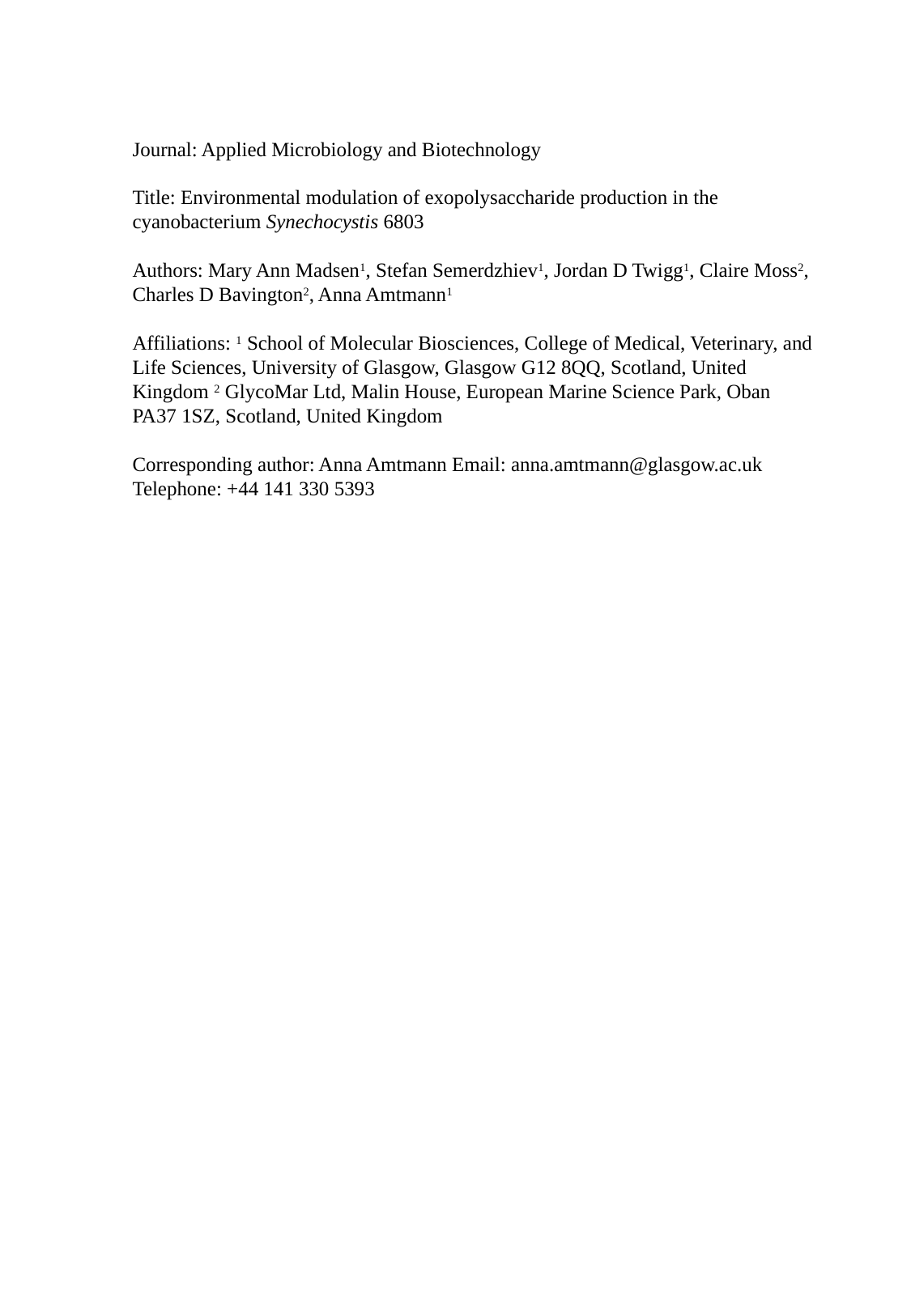

Journal: Applied Microbiology and Biotechnology
Title: Environmental modulation of exopolysaccharide production in the cyanobacterium Synechocystis 6803
Authors: Mary Ann Madsen1, Stefan Semerdzhiev1, Jordan D Twigg1, Claire Moss2, Charles D Bavington2, Anna Amtmann1
Affiliations: 1 School of Molecular Biosciences, College of Medical, Veterinary, and Life Sciences, University of Glasgow, Glasgow G12 8QQ, Scotland, United Kingdom 2 GlycoMar Ltd, Malin House, European Marine Science Park, Oban PA37 1SZ, Scotland, United Kingdom
Corresponding author: Anna Amtmann Email: anna.amtmann@glasgow.ac.uk Telephone: +44 141 330 5393

## Slide 2
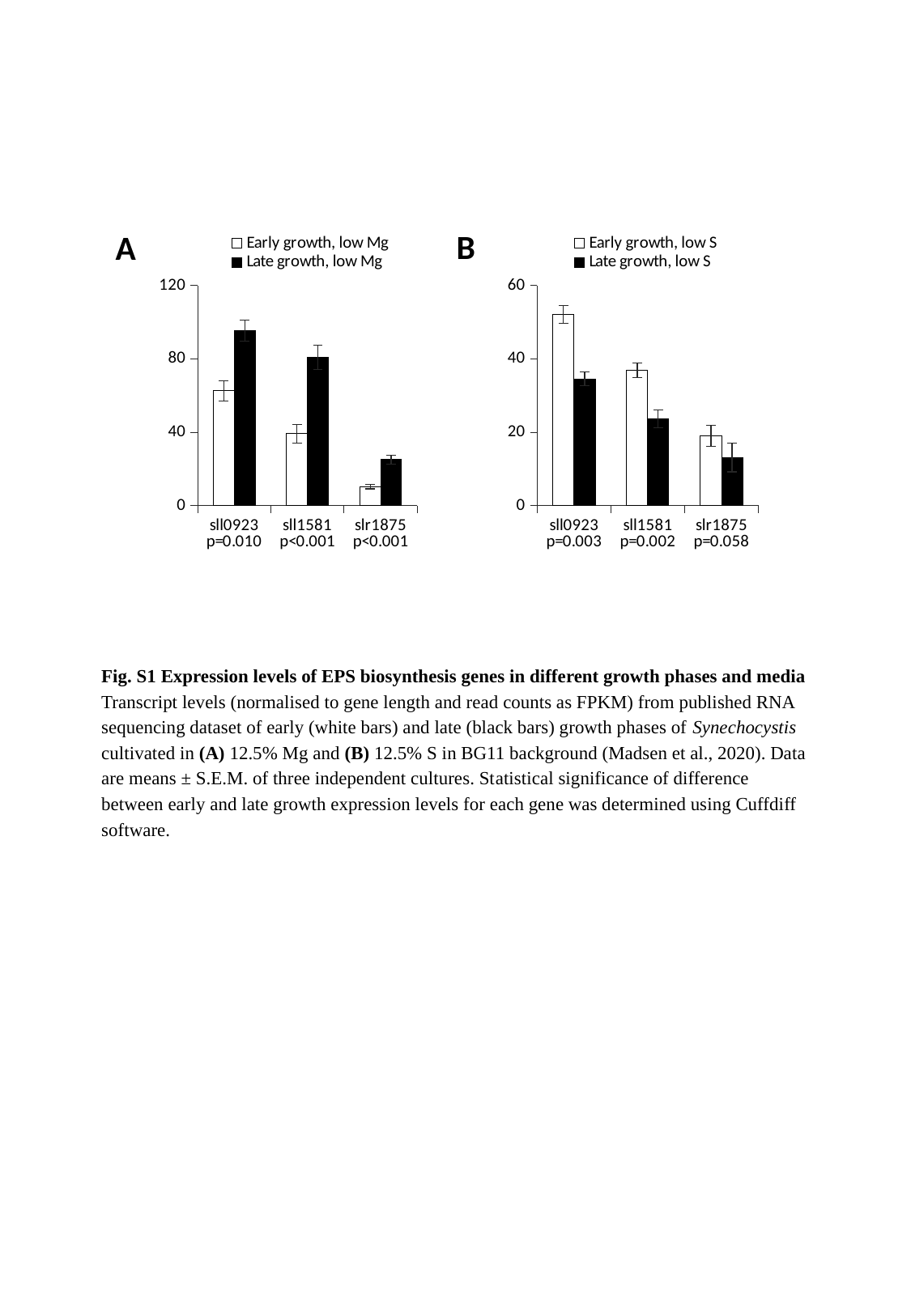

B
A
### Chart
| Category | Early growth, low Mg | Late growth, low Mg |
|---|---|---|
| sll0923
p=0.010 | 62.6424 | 95.42093333333332 |
| sll1581
p<0.001 | 39.229366666666664 | 80.793 |
| slr1875
p<0.001 | 10.380159999999998 | 25.028100000000006 |
### Chart
| Category | Early growth, low S | Late growth, low S |
|---|---|---|
| sll0923
p=0.003 | 52.23193333333334 | 34.55323333333333 |
| sll1581
p=0.002 | 36.91746666666667 | 23.59006666666666 |
| slr1875
p=0.058 | 18.9993 | 13.102456666666667 |Fig. S1 Expression levels of EPS biosynthesis genes in different growth phases and media
Transcript levels (normalised to gene length and read counts as FPKM) from published RNA sequencing dataset of early (white bars) and late (black bars) growth phases of Synechocystis cultivated in (A) 12.5% Mg and (B) 12.5% S in BG11 background (Madsen et al., 2020). Data are means ± S.E.M. of three independent cultures. Statistical significance of difference between early and late growth expression levels for each gene was determined using Cuffdiff software.
