# Supplementary material for: Environmental modulation of exopolysaccharide production in the cyanobacterium Synechocystis 6803
Source: Appl Microbiol Biotechnol. 2023 Aug 8;107(19):6121–34. doi: 10.1007/s00253-023-12697-9 (PMC10485101; doi:10.1007/s00253-023-12697-9)
Supplement: ESM 2 — Fig. S2 Growth and RPS extracts of Synechocystis sp. PCC 6803 in different media (PPTX 212 kb) [file 253_2023_12697_MOESM2_ESM.pptx]

## Slide 1
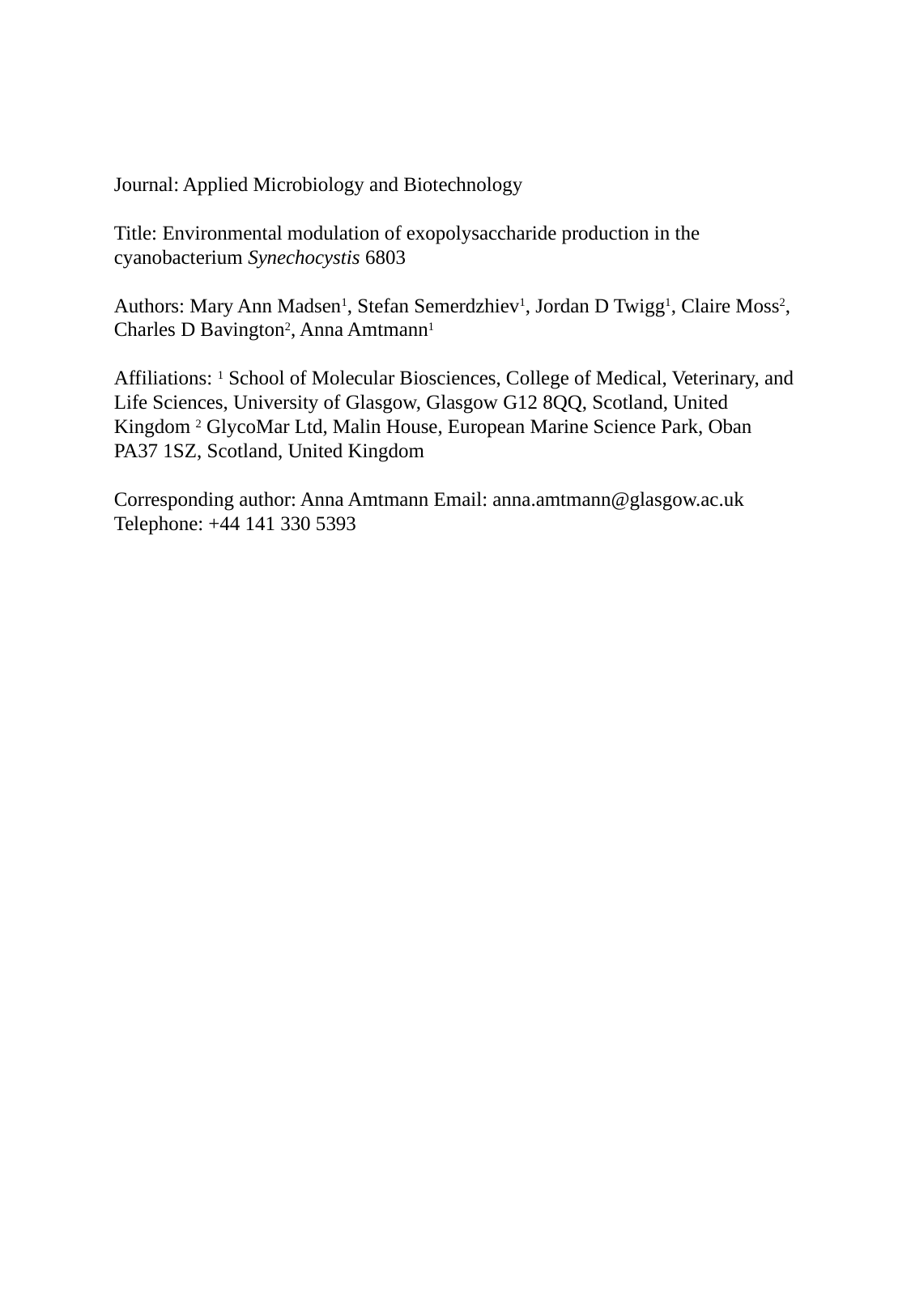

Journal: Applied Microbiology and Biotechnology
Title: Environmental modulation of exopolysaccharide production in the cyanobacterium Synechocystis 6803
Authors: Mary Ann Madsen1, Stefan Semerdzhiev1, Jordan D Twigg1, Claire Moss2, Charles D Bavington2, Anna Amtmann1
Affiliations: 1 School of Molecular Biosciences, College of Medical, Veterinary, and Life Sciences, University of Glasgow, Glasgow G12 8QQ, Scotland, United Kingdom 2 GlycoMar Ltd, Malin House, European Marine Science Park, Oban PA37 1SZ, Scotland, United Kingdom
Corresponding author: Anna Amtmann Email: anna.amtmann@glasgow.ac.uk Telephone: +44 141 330 5393

## Slide 2
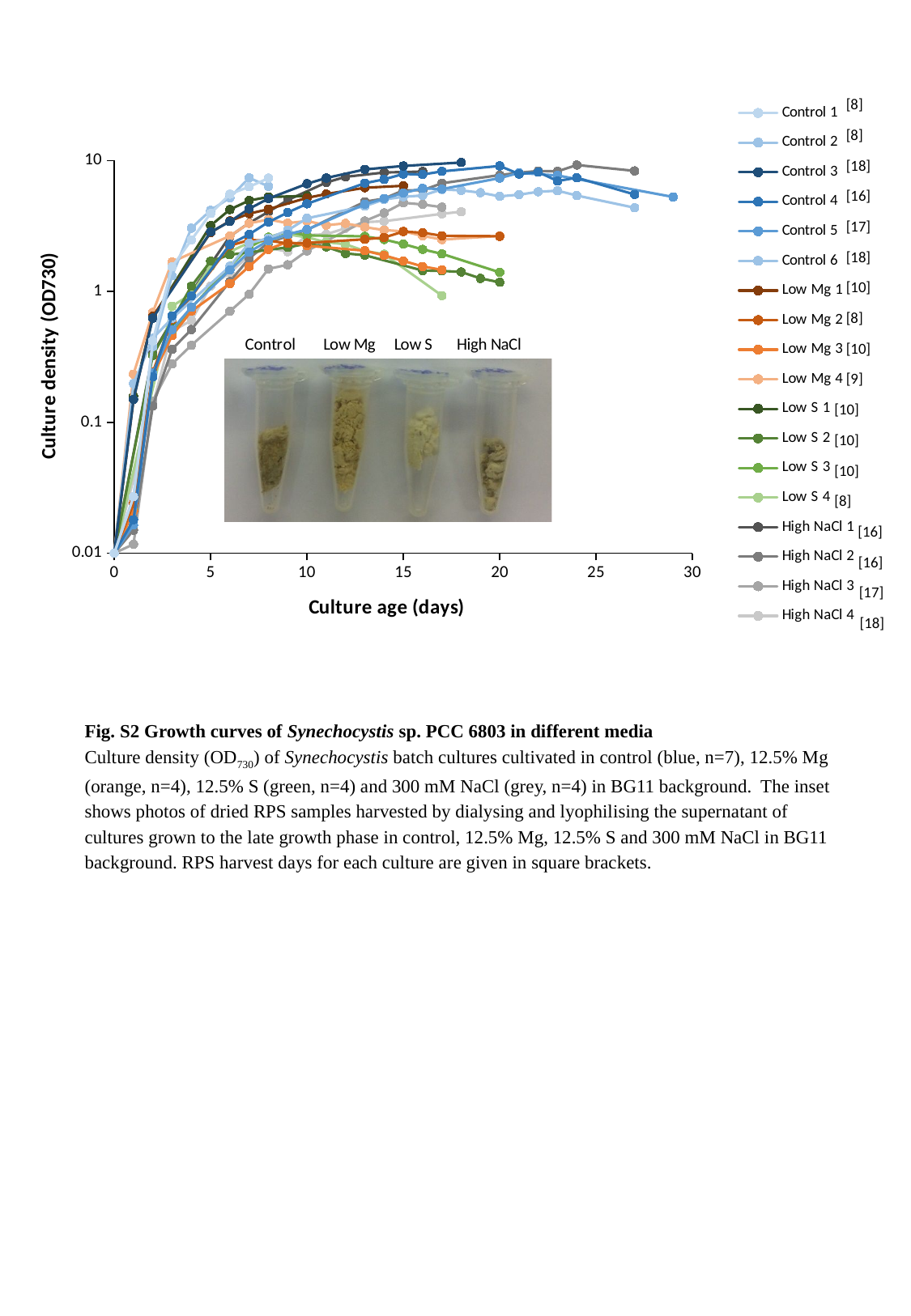

[8]
### Chart
| Category | Control 1 | Control 2 | Control 3 | Control 4 | Control 5 | Control 6 | Low Mg 1 | Low Mg 2 | Low Mg 3 | Low Mg 4 | Low S 1 | Low S 2 | Low S 3 | Low S 4 | High NaCl 1 | High NaCl 2 | High NaCl 3 | High NaCl 4 |
|---|---|---|---|---|---|---|---|---|---|---|---|---|---|---|---|---|---|---|[8]
[18]
[16]
[17]
[18]
[10]
[8]
[10]
[9]
[10]
[10]
[10]
[8]
[16]
[16]
[17]
[18]
Fig. S2 Growth curves of Synechocystis sp. PCC 6803 in different media
Culture density (OD730) of Synechocystis batch cultures cultivated in control (blue, n=7), 12.5% Mg (orange, n=4), 12.5% S (green, n=4) and 300 mM NaCl (grey, n=4) in BG11 background. The inset shows photos of dried RPS samples harvested by dialysing and lyophilising the supernatant of cultures grown to the late growth phase in control, 12.5% Mg, 12.5% S and 300 mM NaCl in BG11 background. RPS harvest days for each culture are given in square brackets.
